# Ten Simple Rules for Approaching a New Job

**DOI:** 10.1371/journal.pcbi.1003660

**Published:** 2014-06-26

**Authors:** Philip E. Bourne

**Affiliations:** Office of the Director, National Institutes of Health, Bethesda, Maryland, United States of America; Whitehead Institute, United States of America

At some point in your professional career, you will be faced with a job interview. This may range from visiting a graduate school where you already have a placement should you want it, to interviewing for a very high-profile position in industry, government, or academia where there is significant competition for that job. Thinking both as a job applicant and a job interviewer about how I have approached job situations over the years before, during, and after the interview and how those situations have turned out, I can offer the following ten simple rules as you prepare. Where appropriate, I conclude a rule with an illustrative scenario for a junior- and/or senior-level position since while the general principles are universal, how they are applied depends somewhat on the seniority of the position.

## Rule 1: Really Want the Job

It is tempting to apply for a job even if you are not sure you want it. As an interviewer, I can say that reading a very generic job application sends a message that the person does not really want the job. This can waste a significant amount of your time as the applicant and the time of those conducting the job search. Chances are you will not get the position because that lack of want will be apparent during one or more interviews—assuming you get as far as an interview. You will lack the passion that the employers are looking for. Everyone, including you, will be disappointed. Be honest with yourself from the outset. Imagine yourself in the job two years in. Is it exactly where you want to be in your career—and life—in two years? Asking yourself whether you really want the job is particularly important if you have been approached to apply for the position. While this is gratifying, remember you are not the only one likely to be asked, and the askers will likely themselves benefit from your application. That benefit for them could be financial in the case of a headhunter approaching you, or more subtle, through improving the asker's reputation if you get the job. Obviously there is more to consider than just the job. A change of job is frequently a life-changing event as well, for example, through relocation, financial change, stress on the family, etc. Making plus and minus columns and discussing the potential job application with all those that it will touch is something that works for me. Then, imagine your life two years into the position and ask the appropriate questions of yourself. Imagine the case of your first tenure-track position, although similar questions apply universally: Am I being productive enough to get tenure? Do I like my work environment and my work colleagues? Am I happy living in this place? What are my future career prospects here?

## Rule 2: Wishful Thinking Is Not Enough—Be Qualified

It is tempting to apply for a position that you are not truly qualified for because you really want it (you have obeyed Rule 1), but deep down you know you are not qualified for it. Beyond the time wasting in applying for something which you have no hope of getting, there is the mental anguish associated with applying for a job. Time is spent wondering, “Will I get it, will I get it?” when that time could be used more productively. Before applying for a job, it is always a good idea to talk to mentors who will give you a candid opinion of your chances before you expend any effort. It may also be helpful to review the qualifications of those in similar positions to determine whether an application makes sense. Having said this, “being qualified” can be a qualitative term. Yes, there will likely be minimal degree requirements, but other aspects of the prerequisite requirements may not be so clear. Years of experience could substitute for a higher degree, relevant experience in a different field might count for something, and so on. Notwithstanding, deep down you will likely know whether you have a chance at a position—be honest with yourself. Again, imagine your first tenure-track application. Do I really have enough publications, grants or promise of grants, teaching experience, and proven service to get this job? Having said all this, it is possible you have a talent or experience that, while not identified in the job posting, really appeals to your potential employer. This is an unusual situation. Be realistic, but at the same time be ambitious—a balance that you will need to judge for yourself.

## Rule 3: Understand and Work the Process

Getting a new job is a process. There is the written application—including cover letter, CV, and possibly a vision or research interest statement of some kind—which you should have someone proofread. Submitting these materials will likely lead to a prescreening, and telephone and in-person interviews may follow. As a reviewer of many such applications, I have to say two things impress me. First, how well the skill set of the applicant maps to the position, and second, how much time the applicant has spent in tailoring the application for the particular position—including their CV. In my opinion, it counts for a lot if the applicant understands the work of the people they will be collaborating with and lays out specifically what they hope to accomplish in working with them. More obvious is the need for the applicant to conform to the process itself—if the application asks for a specific set of skills, outline those skills; more on that in the subsequent rules. You will likely be asked for references as the process progresses. Choose these well. They will likely not be the people who will say the nicest things about you, but people whose opinions are most respected and can provide a value judgment against others in their network. Lastly, the selection process involves a very significant human factor. If, on paper, two applicants appear similar, the one that appeals most to the decision makers will invariably get the job. Think how you can best appeal to the decision makers. Know who those decision makers are (see Rule 8), and as far as possible, what they will be looking for in you as the applicant.

## Rule 4: Be Prepared—Have Something in Writing and Practice the Interview

This works for me and I think would work for most job applicants. Beyond the required documents, I like to map out in writing my thoughts about what I would contribute to the position that is not brought out in the formal application materials. This could be in the form of written answers to imaginary questions that are likely to arise during the interview. By thinking answers through and writing things down ahead of time, you will be less likely to give vague, trite, or at worst, wrong answers to important questions. Questions to address cover the details of the job itself and also questions that arise around many jobs relating to diversity, conflict of interest, ethics, etc. Even better if you can practice the interview with a colleague, or better still with an experienced interviewer. This gets you thinking on the spot and provides instant feedback on how you did. I would even consider videoing the mock interview for later review and diagnosis.

## Rule 5: Do Not Oversell Yourself

This applies both to the written application and any interviews but is more likely to be an issue in an interview situation when you are nervous and eager to impress. Quite simply, do not waffle, fib, or lie (obviously true of the written application too). If you do not know the answer to a question, or feel you do not have a particularly good answer to a question, then say so. While admitting to not knowing something, it is also a good time to indicate you are eager to learn and grow in the new position. Also, if you can't answer a question, request an answer from the interviewer; that will frequently lead to further discussion, which will likely readily indicate you know more than was first conveyed and, again, that you are collegial and willing to listen to the opinions of others.

## Rule 6: Do Not Undersell Yourself

If you come out of the interview thinking, “Damn, I forgot to mention so and so,” then you were likely underprepared and undersold yourself, unless you happen to be well known to the interviewers. Rule 4 is helpful in this regard, since with proper written preparation you will be more likely to give a complete picture of your capabilities. So for example, be prepared to articulate exactly your contributions to your most important and most recent papers. Notwithstanding, in this preparation do not try and learn everything you will need for the job. Getting the job will not depend on what you crammed for the interview, but what experience and knowledge you have acquired over the proceeding years. Make sure that knowledge and experience comes across.

## Rule 7: Understand Your Potential New Workplace

This is important not just by way of helping you decide whether you want to go and work there—there is nothing worse than working in a toxic environment—but also in getting a job offer in the first place. It is all part of doing your homework for the position. This is more than just a web search. Use your network of colleagues to get a sense of the workplace. However, if those colleagues are in the institution to which you have applied, be careful not to put them in a compromising position. Having said that, if the opportunity arises, it is valuable to visit your potential new workplace and talk to people outside of the formal interview process. Let us use two specific examples to give this rule some perspective. First, you have a job interview as a new Assistant Professor in a university in a geographic region new to you. Visit the institution and wander around a day early if you can. Understand the institution—what is the student population, how is it distributed, what are the institutional strengths and weaknesses, etc. Understand the department and/or school you would become part of—what is the faculty to student ratio, what is the breadth of the syllabus taught, what is the research strength, what is the organizational structure, etc. Understand what you will be expected to contribute—suggested courses to teach, collaborative research to undertake, etc. Second, you have a job interview as a software engineer in a for-profit company. Be familiar with the products and services of the company, understand the competition, have some ideas of what you can contribute towards improving products or providing new products. Understand the management structure and how you would fit in.

## Rule 8: Understand Your New Colleagues

As an interviewer, I am impressed if the candidate knows something about what I do and how it relates to their application—what can I say, beyond that I am human. I have also seen this overdone, leaving me with the awful impression the job candidate had been stalking me. Like all that is presented here, there is a balance between overdoing and underdoing it; at least be familiar with the interviewer's latest papers. As an interviewer, if the applicant can see how they would fit in with a couple of specific examples, I will be pleased. Again using our Assistant Professor scenario, that would mean what you would like to teach that would complement courses already offered and a couple of specific research ideas that involve specific collaborations with members of the department and/or school you would be entering. As an interviewee, I ask myself, “Can I see myself working with these folks? How do their interactions and body language bode for my own future in this environment?”

## Rule 9: Be Both Assertive and Humble

This is another example of the need to achieve an imaginary balance that is hard to learn except by experience. It's a component of that nebulous part of your personality known as “people skills.” As you advance in your career, this becomes less of an issue, as you by then have a reputation that is known to at least some of those interviewing you, which got you to the interview stage in the first place. Earlier in your career, you are more likely to be unknown and have got the interview on the strength of your written application and CV. In this case, people skills are important. At the very least, you need to leave the interviewer with the impression, “Yes I would like to hire this person.” To me, that implies that the candidate is both gently assertive and, at the same time, humble. I can't begin to describe how to achieve this.

## Rule 10: Follow up

If there are outstanding issues from the application process, particularly the interview, it is wise to follow up with the chair of the interview committee or the individual interviewer. As an interviewer, this sends two messages to me. First, the candidate would seem to really want the job, and second, I have got additional information that will help in an informed decision. I do not like receiving gratuitous follow-up but rather meaningful input into the decision-making process that I did not have thus far. Others disagree, and believe any kind of follow-up thank you is appropriate.

These rules are just simple guides I have found useful. What should be clear is that this is one person's view, and I invite you to add your own comments on what has worked and not worked for you during the job interview process, either as a candidate or interviewer. Consider it a challenge to crowdsource the perfect job application.

